# A feasibility study of dual-task strategy training to improve gait performance in patients with Parkinson’s disease

**DOI:** 10.1038/s41598-021-91858-0

**Published:** 2021-06-14

**Authors:** Bettina Wollesen, Silvan Rudnik, Alessandro Gulberti, Thomas Cordes, Christian Gerloff, Monika Poetter-Nerger

**Affiliations:** 1 Department of Neurophysiology and Pathophysiology, University Medical Center Hamburg – Eppendorf, Hamburg, Germany; 2 Department of Neurology, University Medical Center Hamburg – Eppendorf, Hamburg, Germany; 3 Present Address: Faculty of Psychology and Human Movement Science, Department of Health Science, University Hamburg, Mollerstr. 10, 20148 Hamburg, Germany

**Keywords:** Neurological disorders, Ageing

## Abstract

Gait disorders in patients with Parkinson’s disease (PD) impact their mobility and self-dependence. Gait training and dual-task (DT)-training improve gait quality. This study aims to assess the feasibility of a specific, gradually intensified DT-training for PD patients with a special focus on gait performance under single task (ST) and DT conditions. Correlations to Freezing of Gait (FoG) were examined. 17 PD patients (70.1 ± 7.4 years, H&Y Stadium 2–3, FoG-Q 9.0 ± 5.5) participated in a four-week DT-training (1x/week, 60 min) with progressively increasing task difficulty and number of tasks. Gait performance (spatiotemporal parameters) was assessed during ST and DT conditions. The training improved DT gait performance, especially gait velocity + 0.11 m/s; (F_(2,16)_ = 7.163; *p* = .0171; η^2^part = .309) and step length (+ 5.73 cm). Also, physical well-being and absolved walking distance improved significantly. Correlation analyses of the FoG score at baseline with relative change of gait metrics post-training revealed significant correlations with training-induced changes of step length and improvement of gait velocity. Overall, the developed DT-training was feasible and effective. Further studies should examine the long-term benefits and the optimal setting to achieve the highest impact. The study was registered in the DRKS (ID DRKS00018084, 23.1.20).

## Introduction

Parkinson’s disease (PD) represents a health condition with increasing prevalence in the aging society, with about 6.1 million PD patients around the world^1^ and one percent of the population over 60 years of age^2^.

The vast majority of PD patients develop gait disorders within the first three years after the diagnosis, which gradually worsen in the course of the disease. One particular feature of the Parkinsonian gait disorder is further impairment of the gait performance when performing another task simultaneously like talking, phoning, or performing cognitive tasks while walking^3^ when both tasks have different goals. In previous studies, PD patients showed an impaired gait performance under single task (ST), but particularly under dual task (DT) conditions compared to age-matched controls^4–6^. A recent systematic review reported these higher DT costs under DT walking conditions in PD patients revealing further reduction of gait velocity, step and stride length, increased gait variability and double support time^7^. These gait changes are accompanied by increased concerns of falling, a higher number of falls, related injuries and hospital stays, a higher level of immobility and and a reduced quality of life^8,9^. Therapeutical approaches of the PD gait disorder and Freezing of Gait (FoG) are challenging, an effective treatment requires a multidisciplinary approach including different training strategies^10^.

From a pathophysiological point of view, cognitive-motor interaction is of high importance for the understanding of DT effects on gait and the development of specific training strategies along with a DT motor learning strategy. The basal ganglia represent an interface in which segregated, parallel motor, cognitive and limbic loops are converged in order to optimise a motor program of a motion sequence. Due to dopamine deficiency in the basal ganglia loops, the integration of cognitive and motor information is disturbed. The limited motor-cognitive information processing in PD patients therefore results in inadequate responses to stimuli while moving in complex everyday situations and DT situations^8,11^.

Conventional physiotherapeutic approaches in PD therapy show benefits for muscle strength, balance and gait performance under ST condition, but not for DT condition^12^. In contrast, several studies that used specific DT training found improvement of DT walking and balance performance^13–16^. Moreover, it has been shown that DT training contributes to gait stabilization in PD patients and reduces DT costs^17^.

Nevertheless, the transfer of training effects to everyday situations remains difficult. This may be due to the lack of methodological exercise principles such as progression and task managing strategies in common DT trainings^18^. Task management strategies were assumed to be important to enable a transfer into different, untrained situations^19^. There is also evidence suggesting that training intensity and complexity needs to be adopted to the individual disabilities of the target group^20^. Finally, patients’ clinical characteristics might impact the responsiveness to DT training effects as the cognitive profile of PD patients or the walking performance at baseline^21^. In summary, the ideal conditions for a task managing, cognitive-motor-DT training that aims at enhancing treatment effects on cognitive-motor performance in PD patients have not been sufficientlty clarified. Additionally, little is known about how training intensity and complexity needs to be adopted to the requirements of the target group, especially if there are additional gait features such as FoG^22^. Within this context, FoG is defined as an episodic absence or marked reduction of forward progression of the feet despite the intention to walk^23^. It was reported to worsen with DT^24,25^.

The aim of this study is to develop and assess a DT managing training for patients with PD, which includes coping strategies, gradual progression in task complexity and scope of training. We focus on the feasibility and effectiveness of DT training to improve gait performance for participants with mild to severe FoG under single and dual-task conditions. We hypothesize that DT training improves quality of gait revealed by objective gait analysis in participants with and without FoG.

## Material and methods

### Trial design and participants

This study was performed between November 2017 and August 2018 as an open-label, monocentric feasibility intervention study at the Department of Neurology of the University Medical Center Hamburg-Eppendorf in an outpatient setting. The study included a pre-post design with baseline measurements (one week before training), four training interventions (once a week) and post training measurements one week after the intervention.

Participants were recruited through advertisements in the journal of the German Parkinson society and by the neurological department of the University Medical Center Hamburg-Eppendorf. Inclusion criteria were: (1) diagnosed idiopathic PD, (2) able to walk more than 25 m without pausing, (3) no change in medication in the following six and last four weeks and (4) sufficiency in the German language. Exclusion criteria were (1) self-reported colorblindness, (2) difficulties to discriminate colors and (3) cognitive decline as measured by MoCA scores < 21^26^.

Participants signed an informed consent following the Declaration of Helsinki (Version of 2013). The study was approved by the local ethics commission, the medical Chamber of Hamburg (Germany, PV5281). All methods were performed in accordance with the relevant guidelines and regulations. The study was registered as a clinical trail in the DRKS (ID DRKS00018084, 23.1.20).

### Primary outcome measures: gait performance

In a daily environment e.g., finding the right bus stop at a busy bus station people have to read signs, identifying the right information on the signs and to inhibit or ignore irrelevant information while walking. This means that walking in daily life situations can be regarded as a dual-task scenario. Besides, gait speed must be adapted, accelerated to maximal speed for e.g., getting to the bus in time*.* To follow the idea of the ecologic validity of a test situation the gait analysis was conducted under three different ST and DT walking conditions: (1) walking in a self-selected walking speed, (2) fast walking speed, (3) walking with a visual-verbal STROOP test. The STROOP test integrated the inhibition of irrelevant information.

Within the STROOP test, participants were asked to name the color of a printed word in a well-lit and quite environment. The meaning of the word differed from its color. The six signs of the STROOP test were placed on the right-hand side of the participants at a height of 1.40 m and without defined distance between the signs to avoid a gait rhythm. Participants received one practice trial in each condition and then data were recorded on the second trial. The order of test conditions was randomized. The same randomized order was used for baseline and post-testing. Within a familiarization trial the assessors ensured that the participants were able to discriminate the colors.

Participants were tested and trained on their regular dopaminergic medication. To avoid circadian effects, the training and testing sessions including the post-training assessment took place at the same time of the day.

Spatio-temporal gait analysis was performed. Velocity, cadence, step length, double support time and Coefficient of Variance (CV) of gait variability of the step length difference were analysed. The CV is calculated as follows: the standard deviation divided by the mean multiplied by 100 (SD/MW*100)^27^. Patients walked 6.1 m on a GAITRite System (CIR Systems Inc, Franklin, NJ, USA). The system is an electronic walkway containing a matrix of pressure sensors with a spatial and temporal resolution of 1.27 cm (length/width of sensors) and 120 Hz respectively. The GAITRite system exhibits excellent reliability for most temporal-spatial gait parameters in older subjects (ICCs: 0.82–0.91)^28^.

Within the gait analysis, the weak and the dominant leg was defined. The weak side was defined as the leg that was more affected by PD (reduced step length, high gait variability). The dominant side was defined as the less affected side (larger step length, reduced gait variability).

### Other measures

At baseline and after six weeks, the level of FoG, concerns about falling and sanitary well-being was measured with the Freezing of Gait questionnaire (FoG-Q)^29^, the Fall Efficacy Scale international Version (FES-I)^30^ and the Short Form Health Survey 12 (SF-12)^31^. Furthermore, hand grip strength of both hands was collected as a measure of general force and fitness. It was measured with a hand dynamometer (Saehan Corp., Masan, South Korea) during sitting and attached arms and flexed elbow to 90°.

#### Feasibility outcome

In order to investigate the feasibility, the absolved number of tasks and meters travelled were documented for every session. Furthermore, the subjectively perceived exercise intensity at half time and at the end of every session was determined using the Rated Perceived Exertion-scale (RPE).

### Intervention

The training was based on the DT managing training by Wollesen and colleagues^32^.

The overall aim of the training was to improve the ability to cope with the simultaneous demands of everyday walking situations and to improve coordination, particularly balance and reactivity to distraction from the environment. This means that the participants needed to learn to inhibit irrelevant information. However, it was not clear if this training intervention was feasible for the examined target group. In particular the parameters task complexity and duration were not examined and/or adjusted for the target group. Therefore, the number of sessions was reduced to examine the feasibility.

The intervention was provided as a one-on-one supervision by a trained sports scientist. The training was conducted for four weeks with one session per week for approximately 60 min in the rooms of the Department of Neurology in the University Medical Center Hamburg-Eppendorf. It included everyday motor skills while balancing and walking which was combined with knowledge acquisition about strategies to reduce the risk of falling in ST conditions^13^. After getting familiar with these tasks, DT aspects were integrated and the program focused more on task prioritization, task switching and transfer to everyday situations (e.g. carrying a tray). The tasks became more complex and included visuo-spatial tasks and tasks with an executive function (e.g. reacting to signs in the opposite direction). Overall, there was an individualised progression of the walking distance (1000 m up to 1750 m) as well as an increase of task difficulty and number of included task combinations. In addition, the progression included movement accuracy and time restrictions (e.g. climbing stairs, carrying shopping bags cf. Table 1).

**Table 1:** Main content of the intervention.

Variable	Distance (m)	Tasks (N)	Main training focus
Session 1	1000	40	Movement endurance, coordination, strength training of the lower extremity
Session 2	1250	50	Introduction to DT situations during walking in everyday life, e.g. avoiding obstacles while walking or reacting to instructions while walking
Session 3	1500	60	Advanced exercises in DT situations while walking and mediation of task managing strategies, e.g. focusing on the footstep rolling behavior
Session 4	1750	70	Combining complex DT situations and mediation of coping strategies, e.g. reacting correctly to direction signs by walking into the opposite direction, while walking with shopping bags

### Statistical analysis

The analysis included means and standard deviations to describe the participants characteristics. Normal distribution was verified with Shapiro–Wilk tests. For all gait parameters except for the CV of the baseline Stroop condition for the left leg normal distribution was given. Two-way repeated measure analysis of variance (ANOVA with a 95% confidence interval) was used to evaluate the effects of the DT training on (1) gait performance and (2) the examination of the completed distance and tasks of the training sessions. Post-hoc tests were adjusted with pair-wise comparisons by using Bonferroni-adjusted t-tests.

Pearson’s one-tailed correlations were performed to analyze the relation of the FoG-scores at baseline 1. with relevant gait parameters at baseline to identify dependencies of gait performance and FoG at baseline 2. with training-induced gait changes to assess a potential impact of FoG severity on training responsiveness. All statistical analyses were conducted with SPSS (version 24.0; SPSS, Inc., Chicago, IL) by an independent assessor, who was not involved in the data collection.

### Ethics approval and consent to participate

Participants signed an informed consent following the Declaration of Helsinki (Version of 2013). The study was approved by the local ethics commission, the medical Chamber of Hamburg (Germany, PV5281).

## Results

### Participants

**Figure 1:**
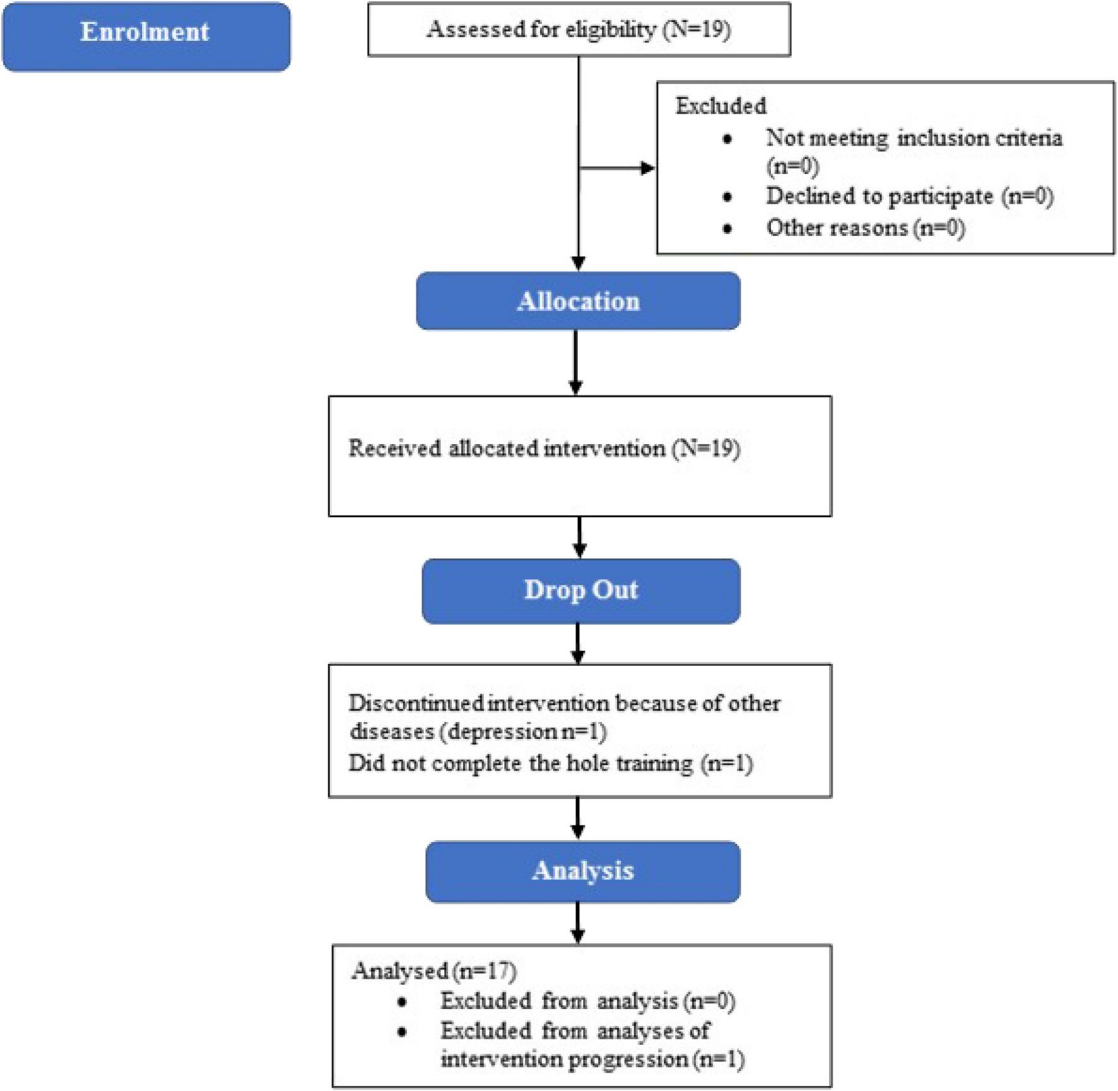
Flow chart of the intervention.

A total number of 19 PD patients were recruited after giving informed consent. One participant could not participate in the study due to increased PD symptoms and depression, a second patient was unable to walk after a fall (due to other diseases; not related to the study program, cf. Fig. 1). 17 participants completed the intervention (mean age 70 ± 7.4 years, 14 males, 3 females). PD patients were in a moderate Hoehn&Yahr stage 2–3. In a subset of PD patients (n = 9), further assessments were available (mean MDS-UPDRS III 24 ± 8.2 with medication, mean disease duration of 8.5 ± 5.0 years, LEDD 1048.4 mg ± 683.4 mg). Most of the PD patients were of the akinetic-rigid subtype (67%), two PD patients had implanted STN-DBS^33^. All patients were tested in everyday conditions with medication and DBS switched on.

Note that for the comparison between the individual intervention progress, 17 patients were taken into account (cf. Table 2).

**Table 2:** Characteristics of patients at baseline (N = 17).

Variables	Total (N = 17)
Age (Years)	70.06 (7.41)
Body masst (kg)	83.18 (15.04)
Body hight (cm)	173.94 (7.77)
BMI (kg/m^2^)	27.36 (3.68)
Hand grip strength left (kg)	31.8 (10.0)
Hand grip strength right (kg)	35.3 (11.4)
H&Y (Stage 1–5)	2.3 (0.6)
FoG-Q (Score of 24)	9.0 (5.5)
FES-I (Score of 64)	23.5 (5.1)
MoCA (Score of 30)	25.5 (2.6)
SF-12 physical (Score of 100)	39.7 (8.6)
SF-12 psychological (Score of 100)	46.2 (9.1)
MDS-UPDRS*	24 (8.2)
Disease duration*	8.5 (5.0)

BMI Body Mass index; H&Y = Hoehn & Yahr Stage; FoG-Q = Freezing of Gait Questionnaire; FES-I = Falls Efficacy Scale International Version; MoCA = Montreal Cognitive Assessment;SF-12 = Short-Form Health Survey – 12.

Note: MDS-UPDRS were evaluated in a limited number of patients (n = 9).

### Training effects on gait performance measures

The comparison of the gait conditions (preferred walking speed, fast walking speed and DT condition) by the two-way ANOVA revealed a significant effect for the three conditions. Within these conditions the DT gait performance revealed impairment of all observed gait parameters compared to the other gait conditions.

All observed gait parameters improved after the training intervention (Table 3).

**Table 3:** Gait Parameters at baseline and post training in the total PD group.

Parameter	Baseline	Post trainng
**ST-preferred walking speed**		
Velocity (m/s)	1.04 (.22)	1.15 (.17)*
Cadence (steps/min)	102.7 (9.0)	107.1 (6.8)
Step length (cm)	60.35 (10.99)	64.80 (9.70)**
Double support time (s)	.343 (.069)	.309 (.037)
%CV step length left leg	5.22 (2.09)	3.84 (2.58)
%CV step length right leg	5.91 (4.60)	3.56 (1.81)
Step length dom (cm)	61.71 (10.58)	66.57 (9.24)**
Step length weak (cm)	59.00 (11.49)	63.04 (10.4)**
**ST-fast walking speed**		
Velocity (m/s)	1.38 (.32)	1.49 (.20)
Cadence (steps/min)	123.6 (15.3)	120.5 (10.9)
Step length (cm)	69.02 (16.73)	74.30 (10.58)
Double support time (sec)	.253 (.064)	.235 (.038)
%CV step length left	9.09 (15.94)	4.61 (3.52)
%CVstep length right	10.11 (20.83)	3.86 (1.91)
step length dom (cm)	70.20 (16.77)	75.65 (11.00)*
Step length weak (cm)	67.84 (16.82)	72.95 (10.48)*
**DT- preferred walking speed**		
Velocity (m/s)	.86 (.27)	.98 (.27)*
Cadence (steps/min)	100.3 (17.7)	101.4 (16.8)
Step length (cm)	51.94 (14.12)	57.67 (11.60)*
Double support time (sec)	.420 (.144)	.397 (.140)
%CV step length left leg	13.19 (19.50)	7.38 (5.45)
%CV step length right leg	14.27 (19.74)	10.83 (8.94)
Step length dom (cm)	54.00 (13.91)	59.45 (11.21)*
Step length weak (cm)	49.88 (14.64)	55.89 (12.50)*

The bold values indicate a *p*-value < 0.05.

*p < 0.05; **p < 0.01.

For gait variability (%CV) = Coefficient of Variation of step length (CV) following formula was used: %CV = (standard deviation/mean) × 100.

The primary endpoint ‘gait speed’ increased after training during preferred walking speed condition as well as under DT and speed conditions by 0.11 m/s (F_(2,16)_ = 7.163; *p* = 0.0171; η^2^part = 0.309). This was accompanied with an increase of cadence by 4.43 steps/min for ST and 1.14 steps/min for DT conditions (cf. Table 3). Within the speed condition cadence decreased by 3 steps/per min (significant time x condition effect (F_(2,15_ = 3.717; *p* = 0.049 η^2^part = 0.331). Also the mean step length increased within in all gait conditions (ST preferred walking speed + 4.45 cm, fast walking: + 5.28 cm, DT: + 5.73 cm, F_(1,16)_ = 9.985, *p* = 0.006, η^2^part = 0.384).

The gait variability of the step length decreased from the initial measurement to postinterventional measurement during preferred walking speed, fast walking and walking under DT conditions in both legs, however the obeseved changes were not significant (Table 3). The mean step length of both the dominant and weak leg, increased as a result of the training under all gait conditions (dominant: *F*_(1,16)_ = 9.683; *p* = 0.007*;* η^2^part = 0.377; weak *F*_(1,16)_ = 9.941; *p* = 0.006*;* η^2^part = 0.383; cf Table 3). The step length of the more affected side increased by an average of 4.04 cm during walking with preferred walking speed and by 5.99 cm during the DT condition.

### Impact of freezing severity on responsiveness to training

In a first step, correlation analyses of FoG-scores with gait metrics at baseline were performed to reveal relevant interactions between FoG severity and gait quality. The FoG score was negatively correlated with the walking speed (preferred walking speed: r = − 0.735; *p* = 0.001; fast speed: r = − 0.667; *p* = 0.003; DT: r = − 0.559; *p* = 0.011), meaning that a higher FoG score was associated with lower gait speed for all conditions. This was accompanied with inverse correlations of the mean step length, CV of step length as well as the mean step length of the more and less affected leg, meaning that higher FoG scores were associated with reduced step length measures. Moreover, there was a significant positive correlation between the FoG scores and the double support time for all conditions (preferred walking speed: r = 0.797; *p* < 0.001; fast speed: r = 0.741; *p* = 0.001; DT: r = 0.656; *p* = 0.004).

**Figure 2:**
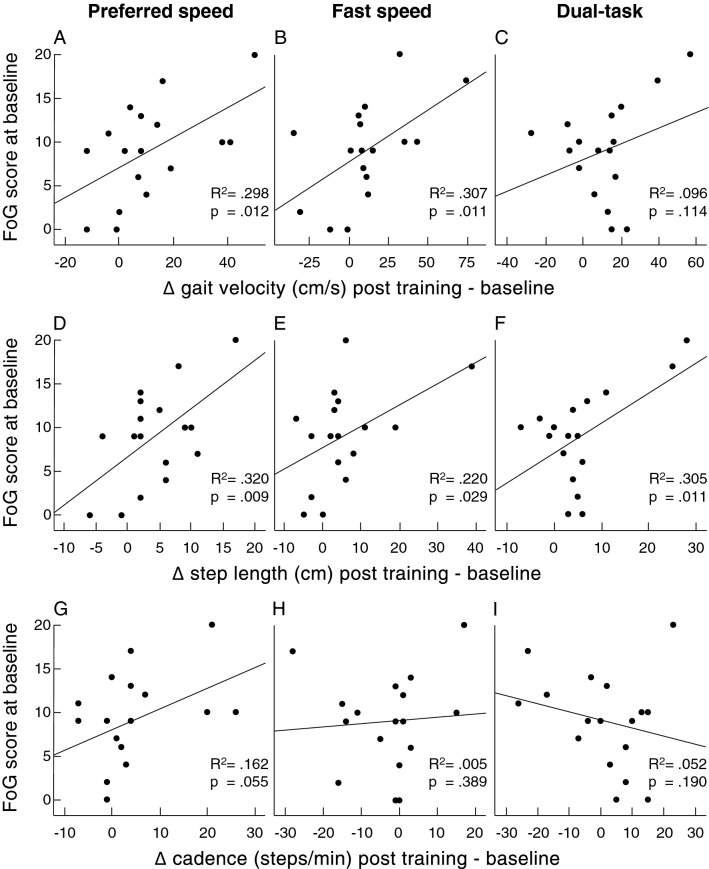
Impact of FOG severity in PD on training responsiveness.

Pearson’s one-tailed correlations of the FoG scores at baseline and the training-induced gait changes for the three walking conditions 1. preferred speed (left column) 2. fast speed (middle column) and 3. DT (right column).The relative gait changes (velocity, first row; mean step length, middle row; cadence, lower row) were calculated as differences of post-training–pre-training gait metrics (cf. Fig. 2).

In a second step, we performed correlation analyses with the FoG score at baseline with the relative change of gait metrics after the training. There were significant correlations with training-induced changes of step length (preferred walking speed: r = 0.566, *p* = 0.018; DT: r = 0.552, *p* = 0.022) and improvement of gait velocity (preferred walking speed: r = 0.546, *p* = 0.012; fast speed: r = 0.554, *p* = 0.011).

### Other outcomes

#### Freezing of gait and grip strength

The total score of the FoG-Q decreased from an average of 9 points to 7.8 points (*F*_*(1,16)*_ = 3.604; *p* = 0.076; η^2^part = 0.184) but failed to be significant. There were no differences between the FoG-groups. During the study period no changes of the hand grip strength in both hands were observed (F_(1,12)_ = 0.218; *p* = 0.649; η^2^part = 0.018; F_(1,11)_ = 0.512; *p* = 0.489; η^2^part = 0.044).

#### Concerns of falling and psychological well-being

The FES-I score for the baseline measurement did not change compared to the output measurement within the two FoG-groups. For the physical SF-12 score there was a significant time effect for the entire cohort. The score increased by 4.1 points from 39.7 to 43.8 points (F_(1,16)_ = 8.166; *p* = 0.011; η^2^part = 0.338). However, no group differences between severe or mild freezers were observed. The psychological score of the SF-12 also increased slightly, but not significantly by 1.2 points (F_(1,16)_ = 0.321; *p* = 0.579; η^2^part = 0.020). There were no differences between the FoG-groups.

#### Feasibility outcomes

The participants were able to raise the completed number of tasks and the amount of walking meters in every training session (F_(3,12)_ = 19.491; *p* < 0.001; η^2^part = 0.830). Post-hoc results revealed that only the improvement from training session three to four was not significant (t (14) = 5.067; *p* = 0.755). There were no significant changes of the RPE scale between the training sessions (F_(3,10)_ = 0.526; *p* = 0.674; η^2^part = 0.136).

## Discussion

The aim of this study was to evaluate the feasibility and beneficial effects of a progressively intensified DT-managing gait training in PD patients, with special regard to patients with FoG. As primary outcome, the study addressed gait speed and walking performance during objective gait analysis. Secondary endpoints were training effects on physical and mental well-being, concerns of falling and FoG. The new DT training strategy induced improvements of gait quality at normal and maximal speed, revealed by an increased gait velocity, step length and reduced gait variability during ST and DT conditions. Patients with severe FoG profited from the DT training. SF-12 and performances within training sessions improved significantly. The hand grip strength remained stable during the intervention period.

These positive results are in line with previous findings by Conradsson and colleagues^34^ who reported an improved gait velocity and step length under ST conditions after ten weeks of training and a total number of 30 training sessions. In addition, the exercise studies by Strouwen and colleagues^35^ as well as the research group around Yogev-Seligmann^3^ were able to improve gait performance under DT condition with their training intervention. With a total amount of 0.11 m/s these improvemnts can be regarded as a meaningful change [cf.^36^]. These effective training interventions had a duration of 25 min for each of the three sessions per week for four weeks. The results of our training strategy is also comparable to the "HiBalance" training by Conradsson et al.^34^ with a duration of ten weeks. With our training intervention that included task managing strategies we were able to gain comparable results with a smaller amount of training time indicating increased efficacy compared to previous training programs. Nevertheless, due to the missing control group these findings can not be generalized.

This study with a gait training of only four weeks is in line with the results of Brauer & Morris^37^ and Conradsson & colleagues^34^. Brauer and Morris^37^ showed that a one-time training of 20 min can gain the same positive effects as the ten-week training of Conradsson et al.^34^ with three units per week, training frequency and intensity might not be the main aspects for postinterventional gait improvement. It seems to be more important to integrate DT managing strategies especially task prioritization and task switching. This hypothesis is supported by results of Fok and colleagues^38,39^ who in accordance to Brauer and Morris^37^ applied two single session of DT trainings which only differed in task managing strategies. When the task prioritization strategy was used, the participants displayed larger improvements of stride length and gait speed under DT conditions compared to ST conditions. Interestingly, the divided attention strategy did not result in any differential effects between ST and DT gait performance^38,39^.

In contrast to study results by Geroin and colleagues^40^ our training intervention addressed task managing strategies and reduced gait variability under DT conditions. In line with Yogev-Seligmann^3^ who especially focused on gait variability, we were able to find positive adaptions, even after four weeks of training. Previous results by Hausdorff^41^ showed that different or only overlapping neural networks may be responsible for gait variability as for gait speed and stride length. The latter may require separate training or a longer training intervention, due to the increased resistance to training. Moreover, our study did not control for the cognitive DT costs. This might have been beneficial for the interpretation of the DT gait results, as there might have been a detrimental effect on cognitive performance. Therefore, these aspects need to be addressed to further develop the program for future interventions.

Interestingly, we observed different training responsiveness of PD patients with different severity of FoG. There were significant correlations with training-induced changes of step length and improvement of gait velocity. Participants with higher freezing scores started at a slower walking speed, probably due to reduced physical fitness, but improved strongly by the training intervention. It has to be noticed that patients with severe freezing in general, i.e. not in the current sample, are older and have had PD for a longer period of time^42^. All participants of this study benefitted in terms of their DT walking performance regardless of FoG severity, the walking speed, gait variability and the step length improved. This underlines the importance of a tailored physical DT training as a therapeutic approach in freezing PD patients.

Besides the gait performance, the physical well-being of all participants improved over the training period. It is assumed that this is a result of the positive experiences during the training sessions^43^ or in general a positive outcome of being physically active^44^. The FoG-score decreased by 1.2 points, however, this change is not significant or clinically meaningful as according to expert clinician rating significance for improvement in the FoG-score is three scale points^45^.

The analysis of feasibility revealed that the number of completed exercises including the increased task complexity of the DT conditions as well as the absolute gait ranges could be significantly increased within four weeks. This means that at first glance the progression of the program was suitable. However, the increase from the third to the fourth training does not show any additional significant progress in the completed exercises and travelled distance. This can be attributed to the excessive simultaneous increase in task difficulty and physical load within the tasks. A milder progression and only an increase in physical or cognitive load might be more feasible to address the requirements of this target group. On the other hand, the rating of the perceived exertion might express that the number of tasks that can be managed within one hour is limited by the accepted exertion of the participants. Therefore, future studies should evaluate the aspect of progression in the light of accepted exertion with the option to integrate more qualitative responses of the participants into the analysis.

Altogether, we can assume that the training strategy of Wollesen and colleagues^14,32^, which had before been evaluated for older people with concerns of falling^13^ and impaired hearing, was tolerated and accepted by PD patients. Our training intervention did not include strategies to improve fast walking. Therefore, changes in the fast walking speed condition could not have been expected. If future goals of these kind of intervention are to improve fast walking speed as well, this should be explicitly addressed within the training routine.

### Limitations

Next to all the positive effects and strengths of this study, there are several limitations.

Regarding the concerns of falling and the FoG-score, the number of falls within the past twelve or six months should have been reported for a broader global impression of falls.

To secure comparability of the participants, the levodopa equivalent dosage should have been monitored. However, as we aimed to prove the feasibility of this intervention in an outpatient therapeutic setting, each patient joined at their individual dose of normal medication. This aspect should be controlled in future studies: Moreover, it should be determined whether the effects of the proposed intervention on main outcome measures were driven by DT training, physical capacities (endurance/lower limb strength) or social interactions with control group designs.

Regarding the test conditions, the cognitive baseline of the Stroop performance should also be integrated into the study design. This would allow to refer to DT costs and would help to better interprete the improvements of DT gait performance.

Regarding the aspect of freezing of gait, additional studies should also integrate a non-freezing group into their analysis to clarify if there are more differences than observed in our evaluation. Finally, a detraining phase or follow-up measurements are missing, next to active or inactive controls groups. These aspects need to be addressed in future studies in order to generalize the promising feasibility results.

## Conclusion

In summary, the new, gradually intensified DT training strategy with a focus on task management is feasible and efficacious for the treatment of PD gait disorder and might become part of a multidisciplinary treatment approach after adjusting to the following aspects: Future randomized controlled trials should integrate (a) recruiting participants for three groups of FoG (1: no FoG; 2: mild FoG and 3: severe FoG) and integrating FoG-score matched controls, (b) careful control for similar medication, (c) report on falls within the last twelve and six months; (d) control for cognitive DT costs; (e) adding follow-up measurements including detraining phases.

## Data Availability

The data that support the findings of this study are available from the corresponding author upon reasonable request.
